# Early Postnatal Care Utilization and Associated Factors Among Women Who Give Birth in the Last Six Weeks in Hosanna Town, Southern Ethiopia, 2022

**DOI:** 10.1155/2024/1474213

**Published:** 2024-05-02

**Authors:** Sintayehu Worku, Merga Dheresa, Tilahun Ali, Mengistu Lodebo

**Affiliations:** ^1^Department of Public Health, Hosanna Health Sciences College, Hosanna, Ethiopia; ^2^School of Nursing and Midwifery, College of Health and Medical Sciences, Haramaya University, Harar, Ethiopia; ^3^School of Nursing and Midwifery, College of Medicine and Health Sciences, Wollo University, Dessie, Ethiopia; ^4^Department of Midwifery, Hosanna Health Sciences College, Hosanna, Ethiopia

**Keywords:** antenatal care follow-up, Ethiopia, postnatal care

## Abstract

**Background:** The early postnatal period is defined as the first 48 h to 7 days after delivery. The early postnatal visit is especially the most critical time for the survival of mothers and newborns, particularly through early detection and management of postpartum complications. Despite the benefits, most mothers and newborns do not receive early postnatal care services from healthcare providers during the critical first few days after delivery.

**Objectives:** This study is aimed at assessing the prevalence of early postnatal care utilization and associated factors among mothers who gave birth within the last 6 weeks in Hosanna town, Southern Ethiopia, from April 20 to May 30, 2022.

**Method:** A community-based cross-sectional study was conducted in Hadiya Zone, Hosanna town, Southern Ethiopia. A simple random sample technique was used to recruit 403 mothers who had given birth in the previous 6 weeks from a family folder. Data was collected through face-to-face interviews using a standardized questionnaire. Binary logistic regression was used to assess the association between outcomes and explanatory variables, and the strength of the association was interpreted using an odds ratio with a 95% confidence interval. In our study, *p* values of 0.05 were considered statistically significant.

**Results:** The prevalence of early postnatal care utilization among mothers who gave birth within 1 week of the study area was 25.8% (95% CI: 21.7–30.0). No formal and primary educational level of husband (AOR = 0.05, 95% CI: [0.02, 0.16]), antenatal care follow-up (AOR = 2.13, 95% CI: [1.11, 4.1]), length of hospital stay before discharge (≥24 h) (*AOR* = 0.3, 95% CI: [0.16, 0.55]), and information about early postnatal care utilization (AOR = 3.08, 95% CI: [1.72, 5.52]) were factors significantly associated with early postnatal care utilization.

**Conclusion:** In comparison to World Health Organization standards, the study's overall prevalence of early postnatal care utilization was low. Early postnatal care use was significantly associated with antenatal care follow-up, the husband's educational level, knowledge of early postnatal care use, and length of stay at the health institution following birth. As a result, the strength of health facilities is to improve service provision, information education, and communication.

## 1. Introduction

The first 6 weeks following birth, known as the postnatal period, were crucial for both the mother and her child [1]. The majority of postnatal maternal and neonatal deaths occur in the first 24 h after birth, and all postnatal maternal and neonatal deaths occur within the first week after the baby is born. Focusing on the immediate postnatal period is crucial, even though the majority of mother and newborn deaths occur between the time of birth and the first 2 days after delivery [2]. Every year in the world, 289,000 cases of maternal mortality and 2.4 million cases of newborn deaths are reported [3]; nearly 99% of maternal deaths occur in low- and middle-income countries, with 67% occurring shortly after childbirth and within 1 week [4, 5]. According to a study that involved 33 nations, sub-Saharan Africa (SSA) has the highest maternal death ratio (MDR) and the highest newborn death rates (NDRs) [6].

A study conducted on 36 African nations reveals that Cameroon had the highest postnatal care service utilization in central Africa (85.52%), while Chad had the lowest (48.03%); in addition, Zimbabwe had the highest postnatal care service utilization in Eastern Africa (84.17%) [7]. In Abi-Adi, Tigray, Ethiopia, the rate of postnatal care use was 11.9%, and 82% of the mothers did not believe that postnatal care provides benefits or reimbursements for both the mother and the newborn [8]. When and why the care was provided to allow the mothers to use it was not accessible in Africa, including the study area, due to a shortage of qualified healthcare providers [9]. The key factors affecting early utilization of postnatal care services included lack of awareness, residence, culture, marital status, ANC follow-up and delivery at a health facility, and the number of children [10].

From the time the baby is delivered until the full range of postnatal care services is provided, routine assessments are made to categorize, treat, or refer to complications for both the mother and the newborn [11]. Different countries have adopted different techniques regarding PNC service consumption. Creating ubiquitous coverage was one of the strategies. The Ethiopian government approves of this plan for enhancing PNC use services. Over the past few years, a large number of health extension professionals have organized and connected health centers with health posts [12]. Overall, prenatal and postpartum medical care is crucial to the survival and general well-being of both the mother and the child [13].

The Ethiopian Federal Ministry of Health advises scheduling three appointments for early postnatal care, ideally on days 3, 7, and 14 and 4–6 weeks after delivery [14]. According to the Ethiopia Mini Demographic Health Survey (EDHS), 17% of women received postpartum treatment within the prescribed 2 days. Among those who used postnatal checkups, 8% had their checks done within 4 h of delivery, 3% within 24 h, 2% within 1 or 2 days, and 5% from 3 to 41 days. In the South region of the postnatal checkups, 26.6% had their checks done within 4 h of delivery, 2.7% within 24 h, 2.7% within 1 or 2 days, 0.5% from 3 to 6 days, and 0.7% from 7–41 days [15].

Early postnatal care, on the other hand, is critical for women, newborns, their families, and even the nation [16]. Numerous studies have shown that reporting of any postnatal care follow-up is insufficient, and the factors influencing this follow-up are poorly understood. However, data are scarce, particularly on early postnatal care in Ethiopia and the research area. As a result, the purpose of this study was to assess the utilization of early postnatal care and associated factors among mothers who had given birth during the previous 6 weeks in Hosanna town, Hadiya Zone, Southern Ethiopia, in 2022.

## 2. Methods and Materials

### 2.1. Study Setting

From April 20 to May 30, 2022, the research was conducted in the Hadiya Zone in Hosanna town, Southern Ethiopia. It is the administrative center for the Hadiya Zone of the Southern Nations, Nationalities, and Peoples' Region, located 194 km from Hawassa and 232 km southwest of Addis Ababa. According to Hadiya Zone Health Department figures for 2018–2019, there were 25,709 people of reproductive age in the town, with around 3820 pregnancies. The government has eight offices for urban health extension workers, three health centers, and one hospital in the town. There are 35 pharmacies, 22 primary clinics, 19 medium clinics, 2 dental clinics, and 2 eye clinics run by private companies [17].

### 2.2. Study Design

A community-based cross-sectional study was employed.

#### 2.2.1. Population

##### 2.2.1.1. Source of Population

The source population of this study is all women who gave birth within the last 6 weeks in Hosanna town, Southern Ethiopia.

##### 2.2.1.2. Study Population

Randomly selected women who gave birth within the last 6 weeks in Hosanna town, South Ethiopia.

#### 2.2.2. Inclusion and Exclusion Criteria

##### 2.2.2.1. Inclusion Criteria

All mothers who had live births within the previous 6 weeks and those who had lived in the town for at least 6 months were included in the study.

##### 2.2.2.2. Exclusion Criteria

Severely unwell or mentally incapable postpartum mothers were excluded from the study during the data collection period.

### 2.3. Sample Size Determination

The sample size was determined by using a single population proportion formula, and the prevalence of early postnatal care utilization was 51.24% [18]. As a result, using the single population proportion calculation, a 95% confidence interval, 5% of marginal error, and 5% of nonresponse rate, the sample size is

 $$\begin{array}{c} n=\frac{{\left(Z\alpha /2\right)}^{2}p\left(1-p\right)}{d^{2}} \end{array}$$

where *n* is the sample size, *Z* is the standard normal value at 95% CI is 1.96, *p* is the prevalence of early postnatal care utilization, and *d*^2^ is the possible margin of error tolerated which is 5%.

 $$\begin{array}{c} \left(1-0.5124\right)=0.4876 \end{array}$$

Then, adding a 5% nonrespondent rate, the final sample size is

$$\begin{array}{cc} \; & Nf=\left(384\right)\;\left(0.05\right)\\ Nf=384+21=405 \end{array}$$

From data (registration books) kept by health posts with the assistance of health extension workers over 3 months, 755 mothers gave birth within the previous 6 weeks.

#### 2.3.1. Sampling Procedure

**Figure 1:**
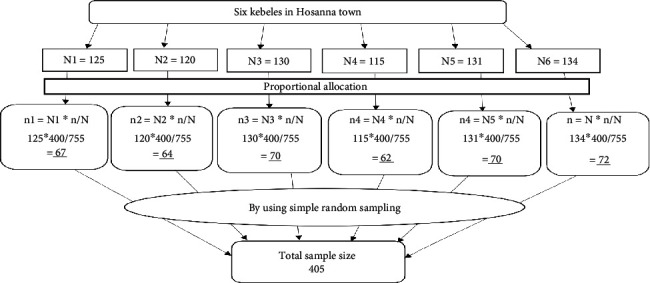
Schematic representations of the sampling technique were employed to assess the usage of early postnatal care and related factors among women who gave birth within the previous 6 weeks in Hadiya Zone, Hosanna town, South Ethiopia, from April 20 to May 30, 2022.

Mothers who gave birth within the previous 6 weeks were chosen using simple random sampling. First proportional allocations of sample size were done to each kebeles based on the 3-month performance of each kebeles. Then, participants were identified by obtaining official lists (reports) from health extension workers working in the kebeles, who routinely collect data on new postnatal mothers to make a sampling frame. Postnatal women were selected using simple random sampling from the existing sampling frame of postnatal women using a computer-generated random number method. Then, data collectors went to the homes of the participants by using their names and house numbers with the guidance of health extension workers (Figure 1).

### 2.4. Study Variables

#### 2.4.1. Dependent Variable

The dependent variable of this study is the utilization of early postnatal care.

#### 2.4.2. Independent Variable

##### 2.4.2.1. Sociodemographic Characteristics

Age, marital status, educational status, religion, economic status, occupation, and residence are the sociodemographic characteristics of this study.

##### 2.4.2.2. Reproductive and Obstetric Factors

Parity, gravidity, obstetric complication, postpartum complication, mode of delivery, previous history of PNC use, and ANC utilization are the reproductive and obstetric factors of this study.

##### 2.4.2.3. Awareness of Postnatal Care

Awareness of postnatal care is provided for mothers and newborn babies.

##### 2.4.2.4. Healthcare Providers and Facility Factors

Place of delivery, delivery attended, discharge advice, length of stay at a health facility after delivery, distance of the health facility, and institutional structure.

## 3. Operational Definitions

1. *Early postnatal care utilization:* when a mother uses at least one PNC service during the first 7 days after giving birth, regardless of where the baby was delivered [1].
2. *Distance from health facility:* postpartum mothers whose home takes more than 1 h by walk or the remoteness of the health facility from the mothers' home [19].
3. *Awareness of the postnatal danger signs:* at least one postpartum obstetrics danger indicator was mentioned by the mothers [20].

## 4. Data Collection Instruments and Procedure

The mother was questioned using a structured questionnaire that had been modified from earlier, related investigations. The questionnaire was used to collect data on sociodemographic characteristics, obstetric and reproductive factors, healthcare institution and provider factors, awareness of postnatal care, and other aspects that measure early postnatal care utilization. The data was collected by six BSc nurses through an interview-structured questionnaire which is prepared in the local language (Hadiyisa).

## 5. Data Quality Control Measures

Training was given to data collectors and supervisors regarding the objective of the study, the data collection tool, and ways of data collection. Before a week of actual data collection, investigators, supervisors, and data collectors took a 5% pretest in Lemmo Woreda Shurmo kebele with similar characteristics to the study population to ensure the clarity of the questionnaire, and then the necessary modifications and corrections were made to standardize and ensure its validity. Both the principal investigator and supervisors oversaw the data collection process. During data collection, both the principal investigator and data collectors checked the data for its completeness and missing information at each point.

## 6. Method of Data Analysis

The collected data was coded, entered into Epi-data version 3.1, and finally exported to SPSS version 26 statistical software for analysis. Recoding and computing of variables were done as needed. Descriptive statistics such as frequency, percentage, mean, standard deviation, and range were used to summarize the data. Bivariate analysis was done to check the crude association between the outcome and independent variables. All variables in bivariate analysis having *p* value < 0.25 were candidates for multivariable logistic regression. A backward likelihood ratio of logistic regression was performed to identify the factors associated with the utilization of early postnatal care. The model goodness of fit was checked by the Hosmer–Lemeshow test, and the *p* value was found to be 0.72 (> 0.05), which revealed that the model was good. Also, multicollinearity was checked, and the value was 1.8. Odds ratios (AORs) at 95% CI were computed to measure the strength of the association between the outcome and the explanatory variables. A *p* value < 0.05 was considered statistically significant in the study.

## 7. Result

### 7.1. Sociodemographic Characteristics

Three hundred eighty-seven mothers were involved in the study, with a response rate of 387 (96%). The participants' ages varied from 25 to 35 years old for 254 (65.6%), with a mean age of 27.62 years old (SD ± 3.8). In terms of education, 269 (69.5%) of the mothers had secondary or higher education.

Similarly, 261 (67.4%) of the husband's educational level was secondary or higher. One hundred seventy-nine (46.3%) of the mothers worked for the government, while one hundred sixty-three (42.1%) of the women's husbands worked for the government as well. Three hundred forty-one (88.1%) of research participants earned more than 1500 ETB each month (Table S1).

### 7.2. Reproductive and Obstetric Characteristics of the Respondents

**Figure 2:**
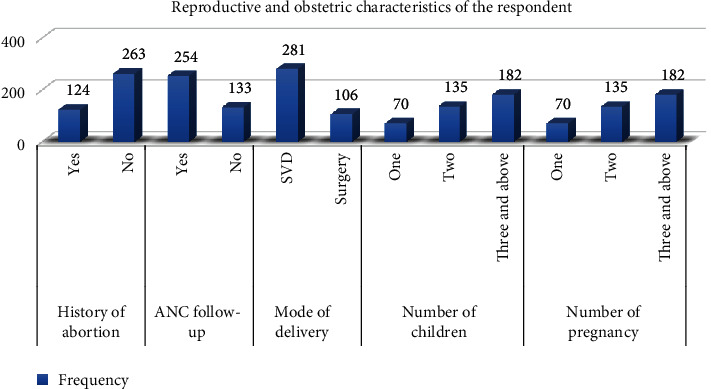
Reproductive and obstetric characteristics of respondents who gave birth in the last 6 weeks in Hadiya Zone, Hosanna town, South Ethiopia, 2022 (*n* = 387).

The majority of respondents (263, 68%) had never had an abortion, and 182 (47%) had three or more children. Out of 387 participants, 169 (43.7%) had two or more pregnancies. In terms of ANC follow-up, nearly three-fourths of 254 (65.6%) had received the follow-up. Around two-thirds of respondents (281, 72.6%) had SVD deliveries (Figure 2).

### 7.3. Obstetric Complications of the Respondents During Pregnancy, at the Time of Delivery, and After Delivery

Thirty percent of the respondents (119) experienced pregnancy-related complications. Thirty-one (26.1%) of the respondents experienced vaginal bleeding. Two-thirds (66.7%) of the mothers had not experienced complications at the time of delivery. Fifty-nine (45.7%) women had a labor that lasted a lengthy period. Nearly three-fourths of respondents (74.2%) experienced no postpartum complications, while 24.8% experienced complications. Slightly less than one-third (32%) of respondents had severe bleeding (Table S2).

### 7.4. Healthcare Provider and Facility Factors in Hadiya Zone, Hosanna Town, Southern Ethiopia, 2022

One hundred eighty-seven (48.3%) of respondents said it took them fewer than 30 min to get to the health facility. One-third of the respondents, 130 (33.6%) and 111 (28.7%), gave birth at government hospitals and health centers, respectively. Three hundred thirty-four (86.3%) of the respondents were attended by health professionals. More than half of them (179, 59.1%) were visited by health extension workers after discharge, and of those, 365 (94.3%) had appointments by health professionals for early postnatal care usage (Table S3).

### 7.5. Awareness of the Mothers on Early Postnatal Care Utilization in Hadiya Zone, Hosanna Town, Southern Ethiopia, 2022

Within 1 week, about 50% (49.4%) of the women were aware of early postnatal care utilization, and 148 (38.2%) mothers received breastfeeding assistance. The biggest problems cited by participants, those mothers who are unable to seek treatment, were cultural pressures. Waiting for the service killed time 172 (44.5%) and lack of information 139 (35.9%) of the mothers, which prevented them from accessing EPNCU within 1 week of delivery (Table S4).

## 8. Prevalence of Early Postnatal Care Utilization

**Figure 3:**
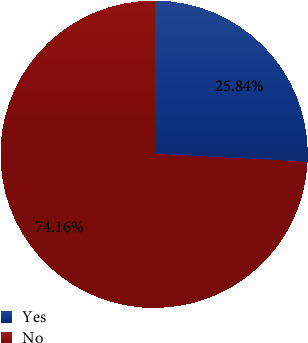
Prevalence of early postnatal care utilization in Hadiya Zone, Hosanna town, Southern Ethiopia, 2022 (*n* = 387).

The prevalence of early postnatal care use within 1 week was determined to be 25.84% (95% CI: 21.7–30.0) in the study area (Figure 3).

## 9. Factors Associated With Early Postnatal Care Utilization

The number of children, information about early postnatal care utilization, mother's occupation, husband's education, ANC follow-up, and length of stay in the medical facility all met the criteria for a minimum *p* value of 0.25 in the bivariate analysis. The outcome of the multivariate analysis revealed that ANC follow-up, husband's educational level (no formal and primary), information about early postnatal care, and length of stay (≥ 2h) in the medical facility after delivery were all substantially associated with the use of early postnatal care.

The study findings showed that mothers who had ANC follow-up were 2 times more likely to use early postnatal care than mothers who had not (AOR = 2.132, 95 CI: [1.110, 4.096]). Also, this study's findings revealed that mothers whose husbands had no formal and primary educational level had a 95% and 85.4% times less likelihood of using early postnatal care than mothers whose husbands had secondary and above (AOR = 0.050, 95% CI: [0.016, 0.158] and AOR = 0.146, 95% CI: [0.065, 0.315], respectively). Similarly, regarding the mother's length of stay in the medical facility after delivery, those who stayed less than 24 h were 70.1% times less likely to use early postnatal care than those who stayed more than 24 h (AOR = 0.299, 95% CI: [0.163, 0.548]). Mothers who had been informed of early postnatal care were 3.082 times more likely to use early postnatal care utilization compared to those who had not been informed (AOR = 3.082, 95% CI: [1.722, 5.520]) (Table S5).

## 10. Discussion

The study revealed that around one-fourth of the respondents were utilizing early postnatal care. Factors like ANC follow-up, information about early postnatal care utilization, the educational level of the husband, length of stay in medical facility after delivery, and mother's awareness were significantly associated with early postnatal care utilization.

The findings of this study showed that EPNCU among mothers who gave birth within the last 6 weeks was 100 (25.8%) (95% CI: 21.7–30.00). This implies that a substantial proportion of postnatal mothers did not use early postnatal care utilization. This study's findings are in line with previous research in Nepal (21.7%) [21], Nigeria (22%) [22], Myanmar (25.20%) [23], and Oromia regional state in Ethiopia (25.3%) [19]. The possible reason might be the socioeconomic standing of the nations, which is the cause of the similarity.

This study finding is lower than the study conducted in Yirgalem town, Sidama Regional State, Ethiopia (45.5%) [24] and Adigrat town, Tigray, Northern Ethiopia (34.3%) [25]. The possible reason for the discrepancy might be, in part, different instruments used, different sample sizes used, and sociocultural and religious differences.

The finding of this study was higher than the result reported from Northwest Tanzania (14.6%) [26], Eastern Uganda (15.4%) [16], Mundri East Country, South Sudan (11.4%) [27], Tigray, Northern Ethiopia (8%) [28], Mertule Mariam, Northwest Ethiopia (19%) [29], secondary analysis of the Ethiopian Demographic Health Survey 2016 (6.3%) [14], and Woango District, South Ethiopia (13.7%) [30]. The possible reason for the discrepancy might be, in part, different sample sizes used, sociocultural differences, and due to the study period in which the study was conducted.

In terms of characteristics related to early postnatal care utilization in this study, mothers who had ANC follow-up were 2 times more likely to use EPNC who did not have ANC follow-up (AOR = 2.13, 95% CI: [1.110, 4.096]). This was consistent with the study conducted in Gedeo Zone, Ethiopia [10, 30, 31] and Sidama Reginal State [20]. According to studies, the effects of ANC follow-up are strongly associated with EPNCU. A possible explanation is that mothers who attend ANC will have the opportunity to receive health education and counseling from health professionals about the availability and benefits of postnatal care and schedules during their ANC follow-up [32].

There is a significant association between utilization of early postnatal care and information about early postnatal care. Women who have information about early postnatal care were 3.082 times more likely to use early postnatal utilization compared to those who have no information (AOR = 3.082, 95% CI: [1.722, 5.520]). This finding was supported by studies conducted in Jimma zone [33], Myanmar [23], Kenya [34], and Mertule Mariam District, Northwest Ethiopia [29]. This could be because health extension workers and health professionals at the community level and hospital facilities provided early postnatal care instruction.

The husband's educational level was associated with early postnatal care utilization. In this study, mothers whose husbands educational level had no formal and primary education were 95% and 85.4% times less likely to use early postnatal care utilization compared to those participants with husbands who had secondary and above educational levels (AOR = 0.050, 95% CI: [0.016, 0.158] and AOR = 0.146, 95% CI: [0.065, 0.315], respectively). This result was supported by the studies conducted on the analysis of the Ethiopian Demographic Health Survey [14], Jimma zone, Southwest Ethiopia [35], and Nepal [21, 36]. The rationale behind this could be that educated husbands have better communication with their wives and are more likely to discuss the use of PNC services as well as other maternal health services. This may also give their wives more independence.

Mothers who had stayed for less than 24 h were 70.1% times less likely to use early postnatal care utilization compared to those respondents who had stayed more than or equal to 24 h (AOR = 0.299, 95% CI: [0.163, 0.548]). This finding was supported by studies conducted in Addis Ababa, Ethiopia [37], and the East Gojjam zone, Northwest Ethiopia [38]. This may be because health institutions can offer sufficient and excellent treatment, and as a result, women are expected to seek follow-up care for their postpartum needs.

### 10.1. Strengths and Limitations of the Study

#### 10.1.1. Strength

This study was trying to minimize recall bias.

#### 10.1.2. Limitation

Due to the nature of the cross-sectional study design, the cause-and-effect relationship was problematic to determine. The other drawback of this study was difficult to generalize the whole population because it cannot include rural populations. Furthermore, because this study did not include qualitative data, the research did not delve into cultural and religious factors.

## 11. Conclusion

Around a quarter (25.8%) of respondents were utilizing early postnatal care services in the study area. ANC follow-up, information on early postnatal care utilization, husband's educational level (no formal and primary), and length of stay (≥ 2 h) at the health facility after delivery were significantly associated with EPNCU. Strengthening healthcare facilities to improve service delivery, information education, and communication. Encourage antenatal care follow-up as well to increase early postnatal care service utilization in the study area.

## Data Availability

The datasets during the current study are available from the corresponding author on reasonable request.

## Supporting Information

Supporting Information Additional supporting information can be found online in the Supporting Information section. Table S1. Sociodemographic characteristics of the study participants of early postnatal care utilization in Hadiya Zone, Hosanna town, South Ethiopia, 2022 (*N* = 387). Table S2. Obstetric complications of the respondents during the time of delivery and after delivery, who gave birth in the last 6 weeks in Hosanna town, South Ethiopia, 2022 (*n* = 387). Table S3. Healthcare provider and facility factors of the respondent in Hadiya Zone, Hosanna town, Southern Ethiopia, 2022 (*n* = 387). Table S4. Awareness of the mothers and early postnatal care utilization in Hadiya Zone, Hosanna town, South Ethiopia, 2022 (*n* = 387). Table S5. Results of bivariate and multivariate logistic regression analysis conducted on mothers who had children within the previous 6 weeks in Hadiya Zone, Hosanna town, South Ethiopia, 2022 (*n* = 387).
