# Supplementary material for: Early Postnatal Care Utilization and Associated Factors Among Women Who Give Birth in the Last Six Weeks in Hosanna Town, Southern Ethiopia, 2022
Source: J Pregnancy. 2024 May 2;2024:1474213. doi: 10.1155/2024/1474213 (PMC11081751; doi:10.1155/2024/1474213)
Supplement: Supporting Information — Additional supporting information can be found online in the Supporting Information section. Table S1. Sociodemographic characteristics of the study participants of early postnatal care utilization in Hadiya Zone, Hosanna town, South Ethiopia, 2022 (N = 387). Table S2. Obstetric complications of the respondents during the time of delivery and after delivery, who gave birth in the last 6 weeks in Hosanna town, South Ethiopia, 2022 (n = 387). Table S3. Healthcare provider and facility factors of the respondent in Hadiya Zone, Hosanna town, Southern Ethiopia, 2022 (n = 387). Table S4. Awareness of the mothers and early postnatal care utilization in Hadiya Zone, Hosanna town, South Ethiopia, 2022 (n = 387). Table S5. Results of bivariate and multivariate logistic regression analysis conducted on mothers who had children within the previous 6 weeks in Hadiya Zone, Hosanna town, South Ethiopia, 2022 (n = 387). [file 1474213.f1.pdf]

Table 1. Socio-demographic characteristics of the study participants of early postnatal care utilization in Hadiya Zone, Hosanna town south, Ethiopia, 2022(N=387).

| Variables | Categories | Frequencies | % |
|---|---|---|---|
| **Age (n = 387)** | <25 | 118 | 30.5 |
| | 25-35 | 254 | 65.6 |
| | >35 | 15 | 3.9 |
| **Religion (n = 387)** | Orthodox | 106 | 27.4 |
| | Protestant | 241 | 62.3 |
| | Muslim | 40 | 10.3 |
| **Ethnicity (n = 387)** | Hadiya | 245 | 63.3 |
| | Gurage | 40 | 10.3 |
| | Kambata | 51 | 13.2 |
| | Silte | 28 | 7.5 |
| | Amhara | 23 | 5.9 |
| **Educational status of the mother (n = 387)** | No formal education | 58 | 15 |
| | Primary education | 60 | 15.5 |
| | Secondary above | 269 | 69.5 |
| **Husband's educational status (n =387)** | No formal education | 73 | 18.9 |
| | Primary education | 102 | 26.4 |
| | Secondary and above | 212 | 54.8 |
| **Occupation of the mothers (n = 387)** | Daily laborer | 58 | 15 |
| | Housewife | 86 | 22.2 |
| | Merchant | 64 | 16.5 |
| | Employee | 179 | 46.3 |
| **Husband occupation (n = 387)** | Farmer | 39 | 10.1 |
| | Merchant | 108 | 27.9 |

| | | | |
|---|---|---|---|
| | Daily laborer | 77 | 19.9 |
| | Employee | 163 | 42.1 |
| **The family income per/month (n = 387)** | <1000ETB | 17 | 4.4 |
| | 1000-1500ETB | 29 | 7.5 |
| | >1500ETB | 341 | 88.1 |

Table 2. Obstetric complications of the respondents during the time of delivery and after delivery, who gave birth in the last six weeks in Hosanna town south, Ethiopia, 2022(n= 387).

| Variables | Categories | Frequencies | % |
|---|---|---|---|
| **Have you ever faced any complications during pregnancy (n= 387)** | Yes | 119 | 30.7 |
| | No | 268 | 69.3 |
| **Complications faced during pregnancy (n = 119)** | Vaginal bleeding | 31 | 26.1 |
| | Severe headache | 21 | 17.6 |
| | Severe abdominal pain | 24 | 20.2 |
| | Blurring of vision | 15 | 12.6 |
| | Poor fetal movement | 16 | 13.4 |
| | High-grade fever | 12 | 10.1 |
| **Did you have complications at the time of delivery (n = 387)** | Yes | 129 | 33.3 |
| | No | 258 | 66.7 |
| **Complications developed at the time of delivery (m = 129)** | Labor lasting a long time | 59 | 45.7 |
| | Fetal distress | 45 | 34.9 |
| | PIH (pre-eclampsia/eclampsia) | 25 | 19.4 |
| **Have you developed a complication after delivery (n = 387)** | Yes | 100 | 25.8 |
| | No | 287 | 74.2 |

| | | | |
|---|---|---|---|
| **Complications developed after delivery (n = 100)** | Heavy bleeding | 32 | 32.0 |
| | High-grade fever | 7 | 7.0 |
| | Severe headache | 20 | 20.0 |
| | Blurring of vision | 24 | 24.0 |
| | Severe abdominal pain | 17 | 17.0 |

Table 3. Health care provider and facility factors of the respondent in Hadiya Zone, Hosanna town southern, Ethiopia, 2022(n= 387).

| Variables | Categories | Frequencies | % |
|---|---|---|---|
| **Time to reach the health facility (n = 387)** | <30 minute | 187 | 48.3 |
| | 30-60 minute | 109 | 28.2 |
| | >=1hr | 91 | 23.5 |
| **Place of birth (n= 387)** | Health post | 53 | 13.7 |
| | Private clinic | 93 | 24 |
| | Health center | 111 | 28.7 |
| | Gov.t hospital | 130 | 33.6 |
| **Birth attended by (n = 387)** | Health professionals | 334 | 86.3 |
| | Health extension workers | 53 | 13.7 |
| **Appointment to the mother by the birth attendant (n = 373)** | Yes | 358 | 97.9 |
| | No | 15 | 2.1 |
| **Did somebody visit you at your home after delivery? (n = 387)** | Yes | 301 | 77.8 |
| | No | 86 | 22.2 |
| **The mother visited after birth by (n =** | Health professionals | 124 | 40.9 |

| | | | |
|---|---|---|---|
| **303)** | Health extension workers | 179 | 59.1 |

Table 4. Awareness of the mothers and early postnatal care utilization in Hadiya zone, Hosanna town South, Ethiopia, 2022(n= 387).

| Variables | Categories | Frequencies | % |
|---|---|---|---|
| **Awareness of the mothers on EPNCU (n = 387)** | Yes | 191 | 49.4 |
| | No | 196 | 50.6 |
| **What are the types of early postnatal care services provided? *** | Physical examination of the mother | 117 | 30.2 |
| | Physical examination of the baby | 102 | 26.4 |
| | Provision of family planning | 84 | 21.7 |
| | Provision of immunization | 53 | 13.7 |
| | Advising on danger signs after delivery | 101 | 26.1 |
| | Advising on nutrition | 58 | 15 |
| | Advising on hygiene | 96 | 24.8 |
| | Advising on breastfeeding | 148 | 38.2 |
| **Obstacles to utilizing EPNCU*** | Cultural not recommended | 172 | 44.5 |
| | Waiting for falling off baby's cord | 38 | 9.8 |
| | To have enough energy | 2 | 0.5 |
| | Pregnancy was unplanned | 2 | 0.5 |
| | Lack of time | 110 | 28.4 |
| | Lack of guardians for my children | 57 | 14.7 |
| | Waiting for the service kills the time | 158 | 40.8 |

| | | |
|---|---|---|
| Lack of information or advise | 139 | 35.9 |

*more than one response

Table 5. Results of bivariate and multivariate logistic regression analysis conducted on mothers who had children within the previous six weeks in Hadiya Zone, Hosanna Town South, Ethiopia, 2022(n=387).

| Characteristics | Categories | EPNCU | | COR 95% CI | AOR 95% CI | p-values |
|---|---|---|---|---|---|---|
| | | **Yes** | **No** | | | |
| **ANC follow-up** | Yes | 81 | 173 | 2.8(1.616, 4.882) | **2.132(1.110, 4.096)** | **0.023** |
| | No | 19 | 114 | 1 | 1 | |
| **Occupation of the mothers** | Daily laborer | 7 | 51 | 0.326(0.139, 0.765) | 0.431(0.159, 1.169) | 0.098 |
| | Housewife | 21 | 65 | 0.768(0.427, 1.382) | 0.854(0.416, 1.753) | 0.668 |
| | Merchant | 19 | 45 | 1.004(0.537, 1.875) | 0.959(0.433, 2.128) | 0.918 |
| | Civil servant | 53 | 126 | 1 | 1 | |
| **Husband educational level** | No formal | 4 | 69 | 0.090(0.032, 0.256) | **0.050(0.016, 0.158)** | **<0.001** |
| | Primary | 13 | 89 | 0.227(0.119, 0.432) | **0.146(0.065, 0.315)** | **<0.001** |
| | Secondary & above | 83 | 129 | 1 | 1 | |
| **Stay an hour in the health facility** | <24 hours | 32 | 152 | 0.4(0.255, 0.666) | **0.299(0.163, 0.548)** | **<0.001** |
| | ≥24 hours | 70 | 133 | 1 | 1 | |
| **Parity** | One | 24 | 46 | 2.4(1.266, 4.384) | 2.153(0.991, 4.679) | 0.053 |
| | Two | 43 | 92 | 2.11(1.251, 3.559) | 1.854(0.980, 3.507) | 0.058 |
| | Three and above | 33 | 149 | 1 | 1 | |

| | | | | | | |
|---|---|---|---|---|---|---|
| **Do you have information about EPNCU?** | Yes | 71 | 120 | 3.407(2.084, 5.570) | **3.082(1.722, 5.520)** | **<0.001** |
| | No | 29 | 167 | 1 | 1 | |
